# Prevalence of malaria and anaemia among HIV infected pregnant women receiving co-trimoxazole prophylaxis in Tanzania: a cross sectional study in Kinondoni Municipality

**DOI:** 10.1186/2050-6511-15-24

**Published:** 2014-04-24

**Authors:** Vicent P Manyanga, Omary Minzi, Billy Ngasala

**Affiliations:** 1 Unit of Pharmacology and Therapeutics, Muhimbili University of Health and Allied Sciences, Dar Es Salaam, Tanzania; 2 Department of Parasitology, Muhimbili University of Health and Allied Sciences, Dar Es Salaam, Tanzania

**Keywords:** HIV, Malaria, Pregnancy, Anaemia, Co-trimoxazole prophylaxis

## Abstract

**Background:**

HIV-infected pregnant women are particularly more susceptible to the deleterious effects of malaria infection particularly anaemia. In order to prevent opportunistic infections and malaria, a policy of daily co-trimoxazole prophylaxis without the standard Suphadoxine-Pyrimethamine intermittent preventive treatment (SP-IPT) was introduced to all HIV infected pregnant women in the year 2011. However, there is limited information about the effectiveness of this policy.

**Methods:**

This was a cross sectional study conducted among HIV-infected pregnant women receiving co-trimoxazole prophylaxis in eight public health facilities in Kinondoni Municipality from February to April 2013. Blood was tested for malaria infection and anaemia (haemoglobin <11 g/dl). Data were collected on the adherence to co-trimoxazole prophylaxis and other risk factors for malaria infection and anaemia. Pearson chi-square test, Fischer’s exact test and multivariate logistic regression were used in the statistical analysis.

**Results:**

This study enrolled 420 HIV infected pregnant women. The prevalence of malaria infection was 4.5%, while that of anaemia was 54%. The proportion of subjects with poor adherence to co-trimoxazole was 50.5%. As compared to HIV infected pregnant women with good adherence to co-trimoxazole prophylaxis, the poor adherents were more likely to have a malaria infection (Adjusted Odds Ratio, AOR = 6.81, 95% CI = 1.35-34.43, P = 0.02) or anaemia (AOR = 1.75, 95% CI = 1.03-2.98, P = 0.039). Other risk factors associated with anaemia were advanced WHO clinical stages, current malaria infection and history of episodes of malaria illness during the index pregnancy.

**Conclusion:**

The prevalence of malaria was low; however, a significant proportion of subjects had anaemia. Good adherence to co-trimoxazole prophylaxis was associated with reduction of both malaria infection and anaemia among HIV infected pregnant women.

## Background

Combined malaria and HIV infection during pregnancy increases the susceptibility of the pregnant women to the negative effects of malaria suggesting a synergistic interaction between HIV infection and malaria [1,2]. HIV-infected pregnant women have significant alterations in immunity to malaria, which render them more vulnerable to the negative effects related to malaria infection [3,4]. This population experiences consistently more peripheral and placental malaria, higher parasite densities, more febrile illnesses, severe anaemia, and adverse birth outcomes than HIV uninfected women regardless of the parity [2].

WHO has recommended a daily dose of co-trimoxazole among HIV-infected pregnant women during the whole pregnancy period in order to prevent opportunistic infections and malaria [5]. Concurrent administration of SP and co-trimoxazole is not advised to avoid adverse reactions [5]. Tanzania had adopted this policy since the year 2011 [6]. Unfortunately, there is limited information about the effectiveness of daily co-trimoxazole for preventing malaria and its deleterious effects particularly anaemia among HIV infected pregnant women.

This study was carried out in a malaria endemic area characterized by malaria transmission throughout the year [7]. In this area, peripheral parasitaemia among pregnant women can be absent or below the detection limit of the microscopic method [8,9]. To overcome this challenge, we used selected malaria rapid diagnostic tests (MRDT) targeting antigens called histidine rich protein 2 (HRP-2). These antigens are released by red blood cells infected by the malaria parasites; they can be detected in the peripheral circulation even when the parasites are sequestered within the placenta [9,10].

This cross sectional study reports the prevalence of malaria, anaemia and the associated risk factors among HIV infected pregnant women receiving co-trimoxazole prophylaxis in one district in Tanzania.

## Methods

### Study design and study area

The design of the study was a cross sectional. It was conducted between February and April 2013 in eight public health facilities in Kinondoni municipality, Dar Es Salaam. Kinondoni has a hot and humid tropical climate with two rainfall seasons: an intense one observed from the month of March to May, and a mild one in November and December. The average temperature ranges from a minimum of 18.1°C to a maximum of 32.1°C. The average annual rainfall is 1,115 mm. A theoretical model based on data for rainfall and temperature characterizes Kinondoni as an area with stable malaria transmission (holoendemic), with transmission occurring during the entire year [7]. Malaria is the leading cause of both the outpatient visits and inpatient admissions [11].

### Study population

The targeted subjects were HIV infected pregnant women receiving co-trimoxazole prophylaxis for more than four weeks. Sample size for this study was calculated using the formula for cross-sectional study based on the study done in Uganda [12]. In that study; thse prevalence of malaria infection among HIV infected pregnant women using co-trimoxazole prophylaxis (x) was found to be 19%. The formula used was:

$$n=\frac{z^{2}x\left(100-x\right)}{\epsilon^{2}}$$

n = Minimum sample size, z = point on standard normal distribution curve corresponding to significance level of 5% (its value is 1.96), x = previous prevalence of malaria infection among HIV infected pregnant women receiving co-trimoxazole prophylaxis (19%), ϵ = margin of error on x (set at 4%). A total of 420 consented subjects on different trimesters and varied gravidities were enrolled into the study. Pregnant women with sickle cell disease, vaginal bleeding and severe medical conditions were excluded from the study.

### Sampling procedure

Kinondoni municipality has a total of 33 public health facilities; among them two are hospitals, one is a health centre and thirty are dispensaries. Cluster sampling was used for selection of health facilities to be included in the study. The health facilities were divided into three clusters, namely hospitals, health centre and dispensaries. The two hospitals and the health centre were included in the study. Five out of the thirty dispensaries were selected using simple random sampling without replacement technique. Each name of the 30 dispensaries was written on a small piece of paper, and then the paper was folded to ‘a ball like’ figure and put in a mug. The mug was thoroughly shaken and five ‘paper balls’ were randomly picked to select the five dispensaries. Two hospitals involved in the study were Mwananyamala and Sinza. A Health Centre involved was Magomeni; and the five dispensaries were Kambangwa, Tandale, Mburahati, Kimara and Mbezi.

Due to the limited number of HIV infected pregnant women, all the subjects in the selected clusters who met the inclusion criteria were enrolled in the study.

### Data collection methods

A structured interview schedule was used to obtain information on various characteristics of the subjects and supplemented by information from patients' files. These include age, weight, height, gravidity, marital status, education level, employment status, WHO clinical stage, use of insecticide treated nets (ITN), history of episodes of malaria illness, use of iron supplements, use of deworming drugs, anti-retroviral treatment category (prophylaxis or lifelong), duration of zidovudine use. The subjects were then tested for malaria and anaemia. The blood sample was collected for CD4 assay.

### Adherence to co-trimoxazole prophylaxis

Adherence to co-trimoxazole prophylaxis was measured by using an interview schedule adapted from Morisky medication adherence scale (Table 1) [13,14].

**Table 1:** A scale used to determine the adherence level for co-trimoxazole prophylaxis among HIV infected pregnant women

**No.**	**Questions**	**Yes**	**No**
i	Do you sometimes forget to take your co-trimoxazole tablets?	0	1
ii	People sometimes miss taking their medicines for reasons other than forgetting. Thinking over the past 2 weeks, were there any days when you did not take your co-trimoxazole tablets?	0	1
iii	Have you ever cut back or stopped taking co-trimoxazole without telling your doctor because you felt worse when you took it?	0	1
iv	When you travel or leave home, do you sometimes forget to bring along co-trimoxazole tablets?	0	1
v	Did you take your co-trimoxazole tablets yesterday?	1	0
vi	Taking medicines every day is a real inconvenient for some people. Do you ever feel hassled about sticking to your treatment plan?	0	1
vii	How often do you have difficulty remembering to take co-trimoxazole tablets?		
	□ Never/Rarely = 1		
	□ Once in a while = 0.75		
	□ Sometimes = 0.5		
	□ Usually = 0.25		
	□ All the time = 0		
**TOTAL SCORE**			

Note: The total score of >6 was interpreted as Good adherence, 4 to 5.9 as Average Adherence and <4 as Poor Adherence.

### Haemoglobin measurement

Haemoglobin was measured by *HemoCue Hb 201 + ®* machine manufactured by HemoCue AB Ängelholm, Sweden. The tip of the middle finger was cleaned by alcohol swab; then a blood sample was obtained by finger pricking procedure. The *Haemocue 201 + ®* cuvette was filled with blood, excess blood was cleaned from the cuvette and air bubbles were removed. The cuvette was placed in the device tray and the holder was pushed gently into the photometer. The results were recorded from the digital display.

### Malaria testing

Malaria was tested by using *SD BIOLINE Malaria Ag P.f/Pan® MRDT* manufactured by Standard Diagnostics, Inc, Korea. The blood sample was collected by finger prick method after the tip of the middle finger was cleaned by alcohol swab. The test device was placed on a clean, flat surface. About 5 μl of whole blood were added into the ‘sample well’ of respective test devices using a micropipette supplied with the test device. Four drops of assay diluent were added into the ‘sample diluent well’. All the test results were recorded within 30 minutes.

### CD-4 count

The CD-4 Count was done at Mwananyamala Hospital by *BD FACS Count®* Machine (BD Biosciences USA). About 4 ml of whole blood (4 ml) was collected in EDTA collection tubes by standard venipuncture procedure. The collected samples were kept at room temperature (18-25°C) and then transported in a special container. All samples were processed within 30 hours from the time of collection.

### Management of patients

Pregnant women who were diagnosed with malaria or anaemia during the study were managed as per the Tanzania’s Standard Treatment Guidelines through the respective health facilities.

### Data analysis

Data were double-entered, cleaned and analysed using computer software called IBM SPSS Statistics 20 (IBM Corp.). Dependent variables were malaria infection and anaemia. While the independent variables were the levels of adherence to co–trimoxazole prophylaxis. Other independent variables were socio-demographic factors, WHO clinical stages, CD-4 count bands, gravidity, pregnancy age (trimesters), sleeping under insecticide treated bed nets (ITN), history of episodes of malaria illness, use of iron supplements, the use of de-worming agent and duration of AZT use (categorized as <3 months, 3 to <6 months and ≥6 months).

Classification of anaemia was based on the recommendation by WHO [15]; normal (Hb ≥11 g/dl), mild anaemia (Hb = 10-10.9 g/dl), moderate anaemia (Hb = 7-9.9 g/dl) and severe anaemia (Hb < 7 g/dl). CD4 count was categorized according to the four bands of HIV related immunodeficiency proposed by WHO [16]. These include: no significant immunodeficiency (≥500 cells/μL), mild immunodeficiency (350–499 cells/μL), advanced immunodeficiency (200–349 cells/μL) and severe immunodeficiency (<200 μL).

Pearson Chi-square Test and Fischer’s Exact Test were used in the univariate analysis between the dependent and independent variables where applicable. Independent variables which showed a statistical significant difference with the outcome variable by univariate analysis were subjected to multivariate logistic regression to determine the predictors of the outcome. The P-value of < 0.05 was considered significant to provide evidence of significant difference or association.

### Ethical considerations

Ethical approval for the study was given by the Ethical Committee of the Muhimbili University of Health and Allied sciences (MUHAS). Permission from Kinondoni Municipal Council to conduct the study in the health facilities was granted. Information about the study was delivered to the patients and a written consent was obtained before the study was conducted. Confidentiality was ensured to all individuals who participated in the study.

## Results

### Characteristics of the study population

From February to April 2013, a total of 420 HIV infected pregnant women in various trimesters were enrolled into the study. The mean ± SD age of the subjects was 28 ± 5.2 years. Table 2 summarizes sociodemographic characteristics of the HIV infected pregnant women who were enrolled in the study.

**Table 2:** Socio-demographic characteristics of HIV infected pregnant women who were enrolled in the study (N = 420)

**Characteristics**	**Number of respondents(n)**	**%**
***Age group (years)***		
<20	16	3.8
20-34	341	81.2
≥35	63	15.0
***Marital status***		
Single	35	8.33
Cohabiting	69	16.43
Married	316	75.24
***Level of education***		
No formal education	33	7.9
Primary	276	65.7
Secondary	87	20.7
Post-secondary	24	5.7
***Employment***		
Employed	123	29.29
Business/self-employed	188	44.76
Not employed	109	25.95

### Prevalence of malaria

The study revealed a prevalence of malaria of 4.5% (19/420). Table 3 shows the prevalence of malaria, according to selected risk factors. A pattern was seen towards the increase in the prevalence of malaria infection as the levels of adherence to co-trimoxazole prophylaxis decreased from good to poor. Pregnant women with poor adherence to co-trimoxazole prophylaxis were 6.8 times more likely to have a malaria infection as compared to those with good adherence (AOR = 6.81, 95% CI =1.35-34.43 and P = 0.02). The prevalence of malaria was statistically similar in all gravidities (P = 0.563).

**Table 3:** Prevalence of malaria according to selected risk factors among HIV infected pregnant women receiving co-trimoxazole (N = 420)

**Characteristics**	**N**	**Malaria prevalence n (%)**	**P-value**	**AOR**	**95% CI**	**P-value**
***WHO clinical stage***						
Stage I	338	12 (3.6)		1		
Stage II	43	2 (4.7)		0.851	0.171-4.226	0.844
Stage III-IV	39	5 (12.8)	0.031^a^	2.305	0.699-7.597	0.170
CD4 count (cells/μL)*						
≥500	104	4 (3.8)		-		
350-499	85	3 (3.5)		-		
200-349	96	8 (8.3)		^−^		
<200	59	4 (6.8)	0.417^a^	-		
***Pregnancy trimester***						
1st trimester	20	1 (5)		-		
2nd trimester	206	9 (4.4)		-		
3rd trimester	194	9 (4.6)	0.986^a^	-		
***Gravidity***						
Primigravidae	139	6 (4.3)		-		
Secundigravidae	148	5 (3.4)		-		
Multigravidae	133	8 (6)	0.563^a^	-		
***Adherence to co-trimoxazole***						
Good	208	2 (1)		1		
Average	80	4 (5)		3.578	0.611-20.955	0.157
Poor	132	13 (9.8)	0.001^a^	6.806	1.346-34.429	0.02
***ITN use***						
Yes	380	15 (3.9)		-		
No	40	4 (10)	0.096^b^	-		
***ART use category***						
Prophylaxis	288	11 (3.8)		-		
Life long	132	8 (6.1)	0.305^a^	-		

^a^Calculated by Pearson Chi Square, ^b^Calculated by Fischer’s Exact Test, *Results of CD4 count were available for only 344 subjects out of 420 (81.9%).

### Prevalence of anaemia

The prevalence of any anaemia (Hb <11 g/dl) was found to be 54% (227/420); while that of mild-to-moderate anaemia and severe anaemia were 49.3% (207/420) and 5% (21/420) respectively. The overall mean ± SD haemoglobin concentration was 10.3 ± 1.5 g/dl.

Table 4 shows the prevalence of anaemia according to various selected risk factors among HIV infected pregnant women. The prevalence of anaemia increased as the levels of adherence to co-trimoxazole prophylaxis decreased from good to poor. Subjects who had poor adherence were 1.8 times more likely to have anaemia as compared to those with good adherence (AOR = 1.75, 95% CI = 1.03-2.98 and P = 0.041).

**Table 4:** Prevalence of anaemia according to selected risk factors among HIV infected pregnant women receiving co-trimoxazole prophylaxis (N = 420)

**Univariate analysis**				**Multivariate analysis**		
**Characteristics**	**n**	**Prevalence of anaemia n (%)**	**P-value**	**AOR**	**95% CI**	**P-value**
***Malaria infection***						
Yes	19	18 (94.7)		10.363	1.329-80.798	0.026
No (reference)	401	209 (52.1)	0.0001^a^	1		
***History of malaria illness***						
Yes	107	76 (71)		1.746	1.005-3.033	0.048
No (reference)	313	151 (48.2)	0.0001^a^	1		
***WHO clinical stage***						
Stage I (reference)	338	165 (48.8)		1		
Stage II	43	32 (74.4)		3.076	1.458-6.491	0.003
Stage III-IV	37	30 (76.9)	0.0001^a^	2.653	1.184-5.945	0.018
CD4 count (cells/μL)*						
≥500	104	48 (46.2)		-		
350-499	85	45 (52.9)		-		
200-349	96	62 (64.6)		^−^		
<200	59	42 (71.2)	0.005^a^	-		
***Pregnancy trimester***						
1st trimester	20	9 (45)		-		
2nd trimester	206	107 (51.9)		-		
3rd trimester	194	111 (57.2)	0.404^a^	-		
***Gravidity***						
Primigravidae	139	72 (51.8)		-		
Secundigravidae	148	83 (56.1)		-		
Multigravidae	133	72 (54.1)	0.767^a^	-		
***ITN use***						
Yes	380	201 (52.9)		-		
No	40	26 (65)	0.144^a^	-		
***ART category***						
ARV Prophylaxis	288	146 (50.7)		-		
Life-long ART	132	81 (61.4)	0.142^a^	-		
***Duration of AZT use (months)***						
<3	162	93 (57.4)		-		
3-5.9	168	92 (54.8)		^−^		
≥6	90	42 (46.7)	0.253^a^	-		
***Iron supplements***						
Yes	338	185 (54.7)		-		
No	82	42 (51.2)	0.567^a^	-		
***Use of de-worming drug***						
Yes	327	171 (52.3)		-		
No	93	56 (60.2)	0.176^a^	-		
***Adherence to co-trimoxazole***						
Good (reference)	208	92 (44.2)		1		
Average	80	47 (58.8)		1.478	0.848-2.578	0.168
Poor	132	88(66.7)	0.0001^a^	1.752	1.03-2.979	0.039

^a^Calculated by Pearson Chi Square, *Results of CD4 Count were available for only 344 subjects out of 420 (81.9%).

The prevalence of anaemia among subjects with malaria infection was 94.7% (18/19) as compared to 52.1% (209/401) among malaria negative subjects. Malaria infected subjects were 10.4 times more likely to have anaemia as compared to those who had a negative malaria test (AOR = 10. 36, 95% CI = 1. 33–80.8, P = 0.026). Likewise, subjects who had at least one episode of malaria illness during the current pregnancy were 1.8 times more likely to have anaemia as compared with those without a history (AOR = 1.75 95% CI = 1.01-3.03, P = 0.048).

The prevalence of anaemia increased as the HIV/AIDS advanced from lower to higher WHO clinical stages (i.e. from stage I to IV). Pregnant women who were on WHO clinical stage III or IV were 2.7 times more likely to have anaemia as compared to those on WHO clinical stage I (AOR = 2.65, 95% CI = 1.18-5.95 and P = 0.018).

## Discussion

This study shows that the prevalence of malaria infection was 4.5% among HIV infected pregnant women receiving co-trimoxazole prophylaxis. A similar low prevalence was obtained in a previous study that was conducted in Malawi by Kapito-Tembo et al. [17]. In that study, the prevalence of malaria among HIV infected pregnant women who were receiving co-trimoxazole prophylaxis was 2.7% and 5.5% by blood smear and real time PCR method respectively. The Malawian study differs from the present study in the method of malaria diagnosis. Real-time PCR targets parasite DNA and has a higher sensitivity than the MRDT; while the blood smear method by microscopy is less sensitive compared to MRDT [9,10,18].

The prevalence of malaria infection increased as the level of adherence to co-trimoxazole decreased from good to poor. Pregnant women with poor adherence were almost seven times more likely to have a malaria infection as compared to those with good adherence. We could not find any previous study that investigated the association between the malaria infection and levels of adherence to co-trimoxazole prophylaxis among similar subjects. An explanation for the pattern seen in our study could be the fact that the good adherents had higher exposure to co-trimoxazole and therefore were more protected as compared with the poor adherents.

Sleeping under insecticide treated nets (ITN) by the subjects could as well have contributed to the low prevalence of malaria in this study group as it was reported in previous studies [19-21]. ITN are freely available to all pregnant women through a voucher system at antenatal clinics [21]. The prevalence of malaria among the subjects who reported to sleep under an ITN was low at 3.9% as compared to those who did not sleep under an ITN which was 10%. The use of ITN control malaria transmission by creating a barrier between the mosquitoes and people sleeping under them [20,21]. Moreover, the insecticides that are used for treating bed nets kill or repel the mosquitoes; consequently, the numbers of mosquitoes that enter the household and attempt to feed on people inside are reduced as well as shortening their length of life [20,21]. Nevertheless, the finding was statistically not significant. This is consistent with the study by West et al. [22], in which other factors excluding ITN were significantly associated with malaria infection in a community with high and equitable distribution of ITN.

In consistent with previous studies [2,12,17,23], malaria infection was distributed to all the gravidities. The explanation for this outcome is that HIV affects the immune memory mechanism which is responsible for the parity-dependent acquisition of antimalarial immunity in pregnancy and therefore predisposes the secundigravidae and multigravidae to a similar risk of malaria infection as the primigravidae [2].

CD-4 Count and WHO clinical staging are parameters which are used to monitor progression of HIV/AIDS [5,24]. This study could not find an association between the prevalence of malaria infection and low CD4 count or advanced WHO clinical stages; which are known to increase the vulnerability to malaria infection [2]. This could be explained by the impact of malaria control interventions; particularly the use of co-trimoxazole prophylaxis and sleeping under an ITN among HIV infected pregnant women. Kapito-Tembo et al. [17] reported similar findings in Malawi whereby HIV infected pregnant women at all bands of CD4 counts (i.e. <200, 200–499 and ≥500 cells/μL) had a similar risk of malaria infection.

The present study showed that the prevalence of anaemia among HIV infected pregnant women receiving co-trimoxazole prophylaxis was 54%. Previous studies that were conducted in the Dar Es Salaam city reported higher prevalence compared to the present study. Finkelstein et al. [25] reported a prevalence of 83% in 1997, while Mehta et al. [26] reported a prevalence of 73% in 2003. Other studies that were conducted in different countries within Sub-Saharan Africa have reported varied values of the prevalence of anaemia among similar subjects [2,17,27,28]. In Malawi; Kapito-Tembo et al. [17] reported a prevalence of 35.6%; while Nkhoma et al. [28] reported a prevalence of 27.4%. In Nigeria, a prevalence of 83.8% was reported by Uneke et al. [27]. The reasons for these variations are not clear, but may be connected to the complexity and the multifactorial aetiology of anaemia among HIV infected pregnant women in sub-Saharan Africa, including HIV/AIDS itself, malaria, protein and micronutrient deficiency and endemic diseases like hookworm and schistosomiasis [25,27].

Good adherence to co-trimoxazole was associated with reduced prevalence of anaemia. A pattern was seen towards the increase in the prevalence of maternal anaemia as the levels of adherence to co-trimoxazole prophylaxis decreased from good to poor. Poor Adherents were almost 1.8 times more likely to have anaemia as compared with good adherents. This could be due to the fact that, well adherent subjects had more exposure to the co-trimoxazole drug and therefore they were more protected from malaria infection and its sequelae particularly anaemia as compared to the poor adherent one. Walker et al. [29], had previously reported the benefits of co-trimoxazole in reducing morbidities among the HIV infected population; this could similarly explain the reduction in anaemia among good adherents.

In consistent with a previous study by Finkelstein et al. [25], advanced HIV/AIDS clinical stages (clinical stage III or IV) or CD4 Count of < 200 had a strong association with anaemia. The subjects who were on WHO clinical stage III or IV were 3 times more likely to have anaemia as compared with those in the WHO clinical stage I. Similarly, the prevalence of anaemia increased as the CD4 Count decreases among the subjects. This pattern could be explained by the fact that subjects with advanced clinical stages or a low CD4 count had poor immunity against malaria and other infections were therefore more vulnerable to becoming anaemic [3,4]. Furthermore, advanced HIV/AIDS is associated with local diseases along the gastrointestinal tract, which could eventually result in poor absorption of nutrients necessary for the formation of haemoglobin [26].

Low CD-4 count and advanced HIV/AIDS stages reflect the chronicity (prolonged existence) of the disease; therefore another cause of anaemia could also be the state of chronic illnesses [30,31]. The immune response mounted against such infections is required for pathogen clearance, but its persistence can cause collateral damage to the host with the occurrence of anaemia as the major pathology [30]. The inflammation triggers the release of chemicals e.g. hepcidin, that signal the iron regulation mechanism to adopt a defensive mode. This type of anaemia is usually characterized by an imbalance between erythro-phagocytosis and erythropoiesis [30,31].

Similar to previous studies [2,27,28], dually infected (HIV plus malaria infection) pregnant women were at considerably greater risk of anaemia as compared with those with HIV infection alone. HIV infected pregnant women who had positive malaria test were ten times more likely to have anaemia as compared with those who had the negative malaria test. Both HIV (particularly with advanced immunosuppression) and malaria infection are individually known causes of anaemia [2]. Anaemia associated with malaria is caused by hemolysis of the red blood cells and hypersplenism, a condition characterized by the exaggeration of inhibitory or destructive function of the spleen [15].

Pregnant women who had at least one episode of malaria illness during the current pregnancy were nearly two times more likely to have anaemia as compared with those without a history. In consistent with this study, Nkhoma et al. [28] reported a significant association between the number of previous malaria episodes and maternal anaemia; having two or more episodes were associated with increased risk of anaemia. The reason could be the fact that both HIV (particularly with advanced immunosuppression) and malaria infection are individually known causes of anaemia [2].

In disagreement with previous knowledge [32-34]; the present study could not find a significant association between anaemia and zidovudine use. This could be due to the positive role of the antiretroviral drugs and co-trimoxazole in controlling the HIV infection and other morbidities and subsequently outweighed the anaemia inducing effect of zidovudine. Furthermore, the multi-aetiologies of anaemia in HIV infected pregnant women in Sub-Saharan Africa [25] e.g. HIV infection itself, malaria infection, nutrients deficiency and worm infestations could be the reason for the high prevalence in both groups and consequently lack of significant difference. Sinha et al. [35] reported similar findings in a study conducted in India. In that study [35], pregnant women who used zidovudine were surprisingly 70% less likely to be anaemic compared with women not receiving zidovudine.

Similar to a study by Finkelstein et al. [25], anaemia prevalence was higher in both the subjects who used iron supplements and those who did not; likewise to the use of de-worming agents. Lack of significant difference between the groups could be explained by the complexity and multifactorial aetiology of anaemia apart from the iron deficiency or worm infestation among HIV infected pregnant women in Sub-Saharan Africa [25,30,31].

Our study had some limitations. We attempted to control the confounders by the multivariate logistic regression; nonetheless, there were potential residual confounding effects from the unmeasured factors like the presence of other infections or pathological conditions. Secondly, because of the cross sectional design of this study, we had only one point of measurement of both the outcomes and exposure; this made it difficult to make a causal inference from the findings. Thirdly, our results may have potentially been affected by information bias because measurement of adherence to co-trimoxazole prophylaxis was done by a self-reported method and no drug levels in the blood were measured. Likewise, the use of iron supplements, de-worming drugs and sleeping under an ITN were self-reported. However, antenatal records were used to verify prescriptions of medications.

## Conclusions

This study has shown a low prevalence of malaria infection among HIV infected pregnant women using the daily co-trimoxazole prophylaxis; however, a significant proportion of subjects had anaemia. Good adherence to co-trimoxazole prophylaxis was associated with reduction of both malaria infection and anaemia. Routine counselling and provision of health education on the importance of good adherence to medications and the consequences of poor adherence are paramount in addressing the existing problems. Mitigation of advanced HIV/AIDS among pregnant women is also important in combating the problem of anaemia. Approaches like early diagnosis and timely initiation of anti-retroviral treatment could be of benefit. Due to the complexity and multiple etiologies of anaemia; other measures like de-worming, schistosomiasis control and nutritional supplementation should be taken on board.

## Abbreviations

95% CI: 95% confidence interval; AIDS: Acquired immunodeficiency syndrome; ANC: Antenatal clinic; AOR: Adjusted odds ratio; ART: Anti-retroviral treatment; ARV: Anti-retroviral; DNA: Deoxyribonucleic acid; EDTA: Ethylenediaminetetra acetic acid; FACS: Fluorescent activated cell sorting; Hb: Haemoglobin; HIV: Human immunodeficiency virus; HRP-2: Histidine rich protein-2; IPT: Intermittent preventive treatment; MMAS: Morisky medication adherence scale; MRDT: Malaria rapid diagnostic test; PCR: Polymerase chain reaction; pLDH: Plasmodium lactate dehydrogenase; PMTCT: Prevention of mother to child transmission of HIV infection; SD: Standard deviation; SP-IPT: Intermittent preventive treatment by sulphadoxine plus pyrimethamine; SPSS: Statistical package for social sciences; WHO: World Health Organization.

## Competing interests

The authors declare that they have no any competing interests.

## Authors’ contributions

VM participated in study design, data collection, data analysis, data interpretation, initiated the manuscript preparation, and participated in the revision process. OM engineered the study design, coordinated data collection, data interpretation and participated in the process of manuscript writing. BN participated in the study design and interpretation of data and manuscript writing. All authors read and approved the final manuscript.

## Authors’ information

VM: Holder of B.Pharm (Hons): Recently completed a Master’s Program in Hospital and Clinical Pharmacy at Muhimbili University of Health and Allied Sciences.

OM: Senior Lecturer at the Unit of Pharmacology and Therapeutics, School of Pharmacy, Muhimbili University of Health and Allied Sciences.

BN: Lecturer at Department of Parasitology, School of Medicine, Muhimbili University of Health and Allied Sciences.

## Pre-publication history

The pre-publication history for this paper can be accessed here:

http://www.biomedcentral.com/2050-6511/15/24/prepub
